# Advances in Plant-Sourced Natural Compounds as Anticancer Agents

**DOI:** 10.3390/molecules31030542

**Published:** 2026-02-04

**Authors:** Violeta Popovici, Emma Adriana Ozon, Cerasela Elena Gîrd, Dumitru Lupuliasa

**Affiliations:** 1 Center for Mountain Economics, “Costin C. Kiriţescu” National Institute of Economic Research (INCE-CEMONT), Romanian Academy, 725700 Vatra-Dornei, Romania; 2 Faculty of Pharmacy, “Carol Davila” University of Medicine and Pharmacy, 020956 Bucharest, Romania; emma.budura@umfcd.ro (E.A.O.); cerasela.gird@umfcd.ro (C.E.G.); dumitru.lupuliasa@umfcd.ro (D.L.)

Cancer remains a significant global health crisis, being one of the foremost causes of mortality. In 2020, the World Health Organization reported approximately 19.3 million new cases and close to 10 million deaths due to the disease [1]. Current projections indicate a concerning trend; it is anticipated that by 2040, the total number of global cancer cases could escalate to 28.4 million, marking a staggering increase of 47% in just two decades [2]. Notably, transitional countries are expected to bear a disproportionate burden of this increase, emphasizing the urgent need for targeted prevention and treatment strategies tailored to these regions.

While there have been substantial advancements in conventional cancer therapies, including surgery, radiotherapy, and chemotherapy, these approaches are not without their limitations. Patients often experience severe side effects, which can significantly impact their quality of life. Furthermore, the development of drug resistance poses a major challenge, rendering some treatments ineffective over time. High treatment costs also create barriers to access, particularly in low- and middle-income countries, where healthcare resources may be limited. The efficacy of these treatments against metastatic disease, which often spreads to distant organs, remains notably inadequate. As a result, there is growing impetus within the scientific community to explore alternative and complementary therapeutic strategies to enhance patient outcomes [3,4].

Natural products, especially those derived from plant sources, have emerged as a promising avenue for developing novel anticancer agents. The vast structural diversity of these compounds, coupled with their selective cytotoxicity—meaning they can selectively target cancer cells while sparing normal cells—makes them particularly attractive for therapeutic applications. Additionally, many plant-derived compounds exhibit multifaceted mechanisms of action, which may help in overcoming resistance issues commonly associated with conventional treatments [5,6].

Historically, the plant world has provided a robust foundation for drug discovery; approximately 60% of currently approved anticancer agents originate from or are inspired by natural sources [5,7]. Among the principal classes of clinically established plant-derived anticancer compounds are vinca alkaloids, such as vincristine and vinblastine, which are extracted from the periwinkle plant, *Catharanthus roseus* (L.) G. Don [8]. Another notable category includes taxane diterpenoids, exemplified by paclitaxel, derived from the bark of *Taxus brevifolia* Nutt. Camptothecin derivatives sourced from *Camptotheca acuminata* Decne and epipodophyllotoxin lignans obtained from various *Podophyllum* species are also significant contributors to the arsenal of plant-derived anticancer therapies [9,10,11,12]. Landmark discoveries in this field, such as leucovorin (in 1950), followed by carzinophilin (in 1954) and vincristine (in 1963), have validated the potential of these compounds in oncology and paved the way for further research [13].

The continued exploration of the plant world as a source of anticancer agents is essential, as it aligns with the overarching goal of developing safer and more effective treatment options for oncological patients.

Thus, all the above-mentioned aspects underscore this Special Issue, which has brought together the most recent research on the anticancer activity of various plant species and their derived products. New and valuable data have been added to this field by the authors of the six original studies and two comprehensive reviews.

Plant extracts contain numerous bioactive compounds, including alkaloids, flavonoids, terpenoids, phenolic acids, polyphenols, saponins, and glycosides [14]. They often interact synergistically, thereby enhancing their overall potential to exert anticancer effects via multiple biological mechanisms [15]. Due to these various molecular mechanisms, plant extracts have demonstrated effectiveness against various cancer types [16]. Two original studies of our Special Issue investigated the composition, antiradical activity, and anticancer potential of two plant extracts [17,18].

First, Jordanian researchers used Soxhlet extraction at 60 °C to obtain a 70% ethanolic extract of *Laurus nobilis* L. leaves [17]. Phytochemical screening by LC-MS/MS identified 21 secondary metabolites with phenolic structures. *L. nobilis* leaf extract has shown promising selective anticancer properties against several cancer cell lines; specifically, against ovarian cancer (ES2), head and neck cancer (SAS), and colorectal cancer (HT-29). Importantly, this extract showed minimal cytotoxicity against normal fibroblast cells, highlighting its potential for targeted cancer therapy. The mechanism of action involves inducing apoptosis and inhibiting cancer cell proliferation by causing G2-phase cell cycle arrest in SAS cells, preventing progression through the cycle, and leading to S-phase accumulation in ES2 cells, where DNA replication occurs. This selective action against cancer cells, along with their safety profile towards normal cells, is due to the well-known dual redox behavior of phenolic compounds, which underscores the therapeutic potential of *L. nobilis* leaf extract in treating various types of cancer [17].

The second study, conducted in Romania, investigated the hydro-ethanolic extract of *Berberis vulgaris* (L.) cortex (BVE) [18]. Thirty-nine phenolic phytocompounds and berberine (an isoquinoline alkaloid) were identified through UHPLC–HRMS/MS and HPLC-DAD. The antioxidant potential of BVE was rigorously evaluated using three distinct methods, illustrating its remarkable protective effects. In addition to antioxidant properties, the acute toxicity and teratogenic effects of both BVE and berberine sulfate hydrate (BS) on *Daphnia* species were evaluated. The findings indicated that BS displayed significant toxicity after just 24 h of exposure. In contrast, BVE manifested its toxic effects after 48 h, leading to notable embryonic developmental delays and raising concerns about its safety to aquatic organisms. Then, the authors assessed the anticancer effects of BVE and BS across six tumor cell lines (MDA-MB-231 breast adenocarcinoma, SK-OV-3 ovarian adenocarcinoma, G2 hepatocyte carcinoma, PE/CA-PJ49 oral (tongue) squamous cell carcinoma, HT-29 colon adenocarcinoma, and LoVo colon adenocarcinoma), examining their impact after 24 and 48 h of treatment. The MTS assay revealed a pronounced dose- and time-dependent antiproliferative activity, with BS demonstrating superior efficacy compared to BVE. The most substantial reduction in tumor cell viability occurred in breast (MDA-MB-231), colon (LoVo), and oral squamous cell carcinoma (PE/CA-PJ49) lines after 48 h of exposure. Importantly, the BVE effects were predominant in cancer cells, as cytotoxicity was not observed in HUVEC normal epithelial cell lines [18].

Essential oils (EOs) are complex mixtures of various chemical compounds, characterized by volatility, unique aromas, low molecular weight, and density. Currently, the EO components are classified into three primary categories: terpenes and terpenoids, which are the major contributors to their therapeutic effects; phenolic compounds, recognized for their antioxidant properties; and aliphatic compounds, with various functional purposes. Numerous studies have highlighted the pharmacological potential of EOs, including their antiradical, antimicrobial, and antidiabetic properties, as well as their potential in cardiovascular disease prevention [19,20,21,22]. Additionally, there is a promising area for investigating the anticancer and antiproliferative activities of EOs, which could significantly enhance their therapeutic potential [23].

Our Special Issue includes two articles that evaluate the anticancer potential of various essential oils [24,25].

An interdisciplinary research team conducted a complex study on three essential oils from Middle Eastern medicinal plants (*Juniperus excelsa* M. Bieb., *Lavandula vera* DC., and *Salvia fruticosa* (Mill)), identifying their compositions using GC-MS analysis and investigating their cytotoxicity in keratinocytes [24]. The authors selected three lines of keratinocytes (normal cells (HaCat), benign (A5), and low-grade malignant (II4)). Their results showed that all essential oils exhibited significant cytotoxic and antiproliferative effects. However, only two of them, from *L. vera* and *S. fruticosa,* affected only malignant cells, particularly at lower concentrations. This selective action highlights their potential applications in targeted therapies for skin cancer [24].

Another original study had an interesting novel approach. The authors obtained essential oils from white, pink, and orange flowers of *Lantana camara* L. (Verbenaceae) from Lebanon, analyzed their composition, and investigated their antioxidant and antitumor activities on two different breast cancer cell lines (MCF-7 and MDA-MB-231) [25]. The results showed that EOs from white flowers exhibited significant, selective cytotoxicity against both breast cancer cell lines, whereas EOs from orange flowers had the highest antioxidant activity.

Key processes involved in anticancer activities of various phytochemicals include: (a) induction of apoptosis [26], (b) cell cycle arrest [27], (c) inhibition of angiogenesis and metastasis [28,29], (d) modulation of immune responses [30,31], (e) interference with signaling pathways (JAK/STAT, ERK/MAPK, PI3K/Akt, Ras/MAPK, and p53) [32,33], and (f) reversal of epithelial–mesenchymal transition (EMT) and prevent metastasis [34]. Thus, modulating these significant pathways, plant constituents can be used as targeted anticancer agents [35]. Another significant advantage of most plants’ secondary metabolites is their selective cytotoxicity, which offers a promising approach to avoid or diminish the severe side effects of conventional oncological drugs [36,37].

Two original studies investigated the molecular mechanisms underlying the anticancer activity of plant bioactive compounds and their derivatives, on various cancer cell lines [38,39].

Ahmed et al. reported that fucoxanthin, a carotenoid found in various types of seaweed, modulates the expression of genes and proteins in the PI3K-Akt signaling pathway in the triple-negative breast cancer (TNBC) cell lines MDA-MB-231 and MDA-MB-468. This pathway is a key for numerous intracellular processes, including cell cycle progression, apoptosis, cell migration, and cellular proliferation. The ability of fucoxanthin to influence these processes suggests that it may act as a regulator within the PI3K-Akt pathway [38]. By modulating the expression and activity of key proteins in this signaling cascade, fucoxanthin could have potential applications as a targeted therapy for TNBC.

Cui et al. synthesized several cordycepin derivatives with higher bioavailability by introducing fat-soluble groups at the N6 position. They investigated their effects on three tumor cell lines: MCF-7 (breast cancer), HepG-2 (liver cancer), and SGC-7901 (gastric cancer). One of these derivatives (noted as 4a) inhibited the MCF-7 breast cancer cells through the mitochondrial pathway, down-regulating the expression of Bcl-2 protein and up-regulating the expression of p53, Bax, Caspase-3, and Caspase-9 proteins [39].

Moreover, this Special Issue was enriched by two comprehensive reviews that reinforce the current knowledge on two bioactive phytochemicals with significant potential for cancer management—curcumenol and γ-tocopherol. Both papers are listed below.

## Abbreviations

LC	Liquid chromatography
HPLC	High-performance liquid chromatography
GC	Gas chromatography
UHPLC-HTMS	Ultra-high-performance liquid chromatography coupled with High-resolution tandem mass spectrometry
MS	Mass spectrometry
DAD	Diode array detector
BVE	The hydro-ethanolic extract of *Berberis vulgaris* (L.) cortex
BS	Berberine sulfate hydrate
G2-phase	The third phase of the cell cycle
DNA	Deoxyribonucleic acid
S-phase	The phase of DNA synthesis in the cell cycle
EO	Essential oil
PI3K	Phosphatidylinositol 3-kinase
Akt	Protein kinase B
JAK/STAT	Janus kinase/Signal transducers and activators of transcription
ERK	Extracellular signal-regulated kinase
MAPK	Mitogen-activated protein kinase
Ras	Rat sarcoma protein
p53	Tumor suppressor protein
EMT	Epithelial–mesenchymal transition
Bax	Bcl-2-associated X protein
Bcl-2	B-cell lymphoma 2 family proteins that regulate programmed cell death
TNBC	Triple-negative breast cancer

## References

[1] Sung H., Ferlay J., Siegel R.L., Laversanne M., Soerjomataram I., Jemal A., Bray F. (2021). Global Cancer Statistics 2020: GLOBOCAN Estimates of Incidence and Mortality Worldwide for 36 Cancers in 185 Countries. CA Cancer J. Clin..

[2] Siegel R.L., Kratzer T.B., Giaquinto A.N., Sung H., Jemal A. (2025). Cancer Statistics, 2025. CA Cancer J. Clin..

[3] Debela D.T., Muzazu S.G., Heraro K.D., Ndalama M.T., Mesele B.W., Haile D.C., Kitui S.K., Manyazewal T. (2021). New Approaches and Procedures for Cancer Treatment: Current Perspectives. SAGE Open Med..

[4] Quintero-Rincón P., Caballero-Gallardo K., Olivero-Verbel J. (2025). Natural Anticancer Agents: Prospection of Medicinal and Aromatic Plants in Modern Chemoprevention and Chemotherapy. Nat. Prod. Bioprospect..

[5] Cragg G.M., Pezzuto J.M. (2016). Natural Products as a Vital Source for the Discovery of Cancer Chemotherapeutic and Chemopreventive Agents. Med. Princ. Pract..

[6] Naeem A., Hu P., Yang M., Zhang J., Liu Y., Zhu W., Zheng Q. (2022). Natural Products as Anticancer Agents: Current Status and Future Perspectives. Molecules.

[7] Majolo F., de Oliveira Becker Delwing L.K., Marmitt D.J., Bustamante-Filho I.C., Goettert M.I. (2019). Medicinal Plants and Bioactive Natural Compounds for Cancer Treatment: Important Advances for Drug Discovery. Phytochem. Lett..

[8] Khazir J., Mir B.A., Pilcher L., Riley D.L. (2014). Role of Plants in Anticancer Drug Discovery. Phytochem. Lett..

[9] Belete T.M., Beyna A.T. (2025). Review on the Ethnopharmacological Use of Medicinal Plants and Their Anticancer Activity from Preclinical to Clinical Trial. Nat. Prod. Commun..

[10] Shoeb M. (2008). Anticancer Agents from Medicinal Plants. Bangladesh J. Pharmacol..

[11] Xiao J., Gao M., Sun Z., Diao Q., Wang P., Gao F. (2020). Recent Advances of Podophyllotoxin/Epipodophyllotoxin Hybrids in Anticancer Activity, Mode of Action, and Structure-Activity Relationship: An Update (2010–2020). Eur. J. Med. Chem..

[12] Adak D., Ray P., Setua S. (2024). Unlocking Therapeutic Precision: “Camptotheca Acuminata, a Traditional Chinese Herb Tailored for Phytonano-Cancer Theranostics”. Pharmacol. Res.-Mod. Chin. Med..

[13] Izzo A.A., Stefanska B. (2025). Natural Products and Cancer: From Drug Discovery to Prevention and Therapy. Br. J. Pharmacol..

[14] Yuan H., Ma Q., Ye L., Piao G. (2016). The Traditional Medicine and Modern Medicine from Natural Products. Molecules.

[15] Bajpai P., Usmani S., Kumar R., Prakash O. (2024). Recent Advances in Anticancer Approach of Traditional Medicinal Plants: A Novel Strategy for Cancer Chemotherapy. Intell. Pharm..

[16] Lu J.-J., Bao J.-L., Wu G.-S., Xu W.-S., Huang M.-Q., Chen X.-P., Wang Y.-T. (2013). Quinones Derived from Plant Secondary Metabolites as Anticancer Agents. Anti-Cancer Agents Med. Chem..

[17] Al-Mammori F., Qasem A.M.A., Al-Tawalbeh D., Abuarqoub D., Hmedat A. (2025). The Therapeutic Potential of *Laurus nobilis* L. Leaves Ethanolic Extract in Cancer Therapy. Molecules.

[18] Ivan I.M., Olaru O.T., Popovici V., Chițescu C.L., Popescu L., Luță E.A., Ilie E.I., Brașoveanu L.I., Hotnog C.M., Nițulescu G.M. (2024). Antioxidant and Cytotoxic Properties of *Berberis vulgaris* (L.) Stem Bark Dry Extract. Molecules.

[19] Konfo T.R.C., Djouhou F.M.C., Koudoro Y.A., Dahouenon-Ahoussi E., Avlessi F., Sohounhloue C.K.D., Simal-Gandara J. (2023). Essential Oils as Natural Antioxidants for the Control of Food Preservation. Food Chem. Adv..

[20] Pandey A.K., Kumar P., Singh P., Tripathi N.N., Bajpai V.K. (2017). Essential Oils: Sources of Antimicrobials and Food Preservatives. Front. Microbiol..

[21] Orchard A., van Vuuren S. (2017). Commercial Essential Oils as Potential Antimicrobials to Treat Skin Diseases. Evid.-Based Complement. Altern. Med..

[22] Ejiofor E., Oyedemi S., Egedigwe-Ekeleme C., Obike C., Aguwamba C., Nweje-Anyalowu P., Ejiofor M., Okwuonu O. (2023). Essential Oil Components with Antidiabetic and Anti-Obesity Properties: A Review of Mechanisms of Action and Toxicity. J. Essent. Oil Res..

[23] Mohamed Abdoul-Latif F., Ainane A., Houmed Aboubaker I., Mohamed J., Ainane T. (2023). Exploring the Potent Anticancer Activity of Essential Oils and Their Bioactive Compounds: Mechanisms and Prospects for Future Cancer Therapy. Pharmaceuticals.

[24] Othman R., Moarbes V., Zaatar M.T., Antonios D., Roufayel R., Beyrouthy M., Fajloun Z., Sabatier J.-M., Karam M. (2025). Cytotoxic Activity of Essential Oils from Middle Eastern Medicinal Plants on Malignant Keratinocytes. Molecules.

[25] El Hajj J., Karam L., Jaber A., Cheble E., Akoury E., Kobeissy P.H., Ibrahim J.-N., Yassin A. (2024). Evaluation of Antiproliferative Potentials Associated with the Volatile Compounds of *Lantana camara* Flowers: Selective In Vitro Activity. Molecules.

[26] Amirghofran Z., Shekofteh N., Ghafourian M., Khosravi N., Kalantar K., Malek-Hosseini S. (2019). Tumor Cell Death via Apoptosis and Improvement of Activated Lymphocyte Cytokine Secretion by Extracts from *Euphorbia hebecarpa* and *Euphorbia petiolata*. Asian Pac. J. Cancer Prev..

[27] Pumiputavon K., Chaowasku T., Saenjum C., Osathanunkul M., Wungsintaweekul B., Chawansuntati K., Wipasa J., Lithanatudom P. (2017). Cell Cycle Arrest and Apoptosis Induction by Methanolic Leaves Extracts of Four Annonaceae Plants. BMC Complement. Altern. Med..

[28] Lu X., Friedrich L.J., Efferth T. (2025). Natural Products Targeting Tumour Angiogenesis. Br. J. Pharmacol..

[29] Wang Y., Huang M., Zhou X., Li H., Ma X., Sun C. (2025). Potential of Natural Flavonoids to Target Breast Cancer Angiogenesis (Review). Br. J. Pharmacol..

[30] Afshari A.R., Sanati M., Ahmadi S.S., Kesharwani P., Sahebkar A. (2024). Harnessing the Capacity of Phytochemicals to Enhance Immune Checkpoint Inhibitor Therapy of Cancers: A Focus on Brain Malignancies. Cancer Lett..

[31] Lee J., Han Y., Wang W., Jo H., Kim H., Kim S., Yang K.-M., Kim S.-J., Dhanasekaran D.N., Song Y.S. (2021). Phytochemicals in Cancer Immune Checkpoint Inhibitor Therapy. Biomolecules.

[32] Krishnasamy N., Ramadoss R., Niranjan K.C., Amberkar V.S., Sahoo H.C., Devadoss P. (2025). Multiomics and Molecular Docking Approaches to Elucidate *Desmostachya bipinnata*-Derived Multitargeting Agents for Oral Squamous Cell Carcinoma. J. Pharmacol. Pharmacother..

[33] Mitea G., Schröder V., Iancu I.M., Mireșan H., Iancu V., Bucur L.A., Badea F.C. (2024). Molecular Targets of Plant-Derived Bioactive Compounds in Oral Squamous Cell Carcinoma. Cancers.

[34] Nimal S., Kumbhar N., Pote M.S., Bankar R., Shaikh M., Gacche R. (2025). Reversal of Epithelial to Mesenchymal Transition in Triple Negative Breast Cancer through Epigenetic Modulations by Dietary Flavonoid Galangin and Its Combination with SAHA. Cell Commun. Signal..

[35] Geevarghese A.V., Ranganathan H. (2025). Elucidating the Potential of Phytochemicals in Targeting Various Signalling Pathways of Oral Cancer: Current Update on Their Clinical Translational Potential. Pharmacol. Res.-Nat. Prod..

[36] Giordano M.E., Lionetto M.G. (2023). Intracellular Redox Behavior of Quercetin and Resveratrol Singly and in Mixtures. Molecules.

[37] Fernández-Moriano C., Divakar P.K., Crespo A., Gómez-Serranillos M.P. (2017). Protective Effects of Lichen Metabolites Evernic and Usnic Acids Against Redox Impairment-Mediated Cytotoxicity in Central Nervous System-like Cells. Food Chem. Toxicol..

[38] Ahmed S.A., Mendonca P., Messeha S.S., Oriaku E.T., Soliman K.F.A. (2024). The Anticancer Effects of Marine Carotenoid Fucoxanthin through Phosphatidylinositol 3-Kinase (PI3K)-AKT Signaling on Triple-Negative Breast Cancer Cells. Molecules.

[39] Cui L., Zhao L., Shen G., Yu D., Yuan T., Zhang Y., Yang B. (2024). Antitumor Mechanism and Therapeutic Potential of Cordycepin Derivatives. Molecules.

